# A Developmental Switch for Hebbian Plasticity

**DOI:** 10.1371/journal.pcbi.1004386

**Published:** 2015-07-14

**Authors:** Marijn B. Martens, Tansu Celikel, Paul H. E. Tiesinga

**Affiliations:** 1 Donders Institute for Brain, Cognition and Behaviour, Centre for Neuroscience, Department of Neuroinformatics, Radboud University Nijmegen, Nijmegen, The Netherlands; 2 Donders Institute for Brain, Cognition and Behaviour, Centre for Neuroscience, Department of Neurophysiology, Radboud University Nijmegen, Nijmegen, The Netherlands; Research Center Jülich, GERMANY

## Abstract

Hebbian forms of synaptic plasticity are required for the orderly development of sensory circuits in the brain and are powerful modulators of learning and memory in adulthood. During development, emergence of Hebbian plasticity leads to formation of functional circuits. By modeling the dynamics of neurotransmitter release during early postnatal cortical development we show that a developmentally regulated switch in vesicle exocytosis mode triggers associative (i.e. Hebbian) plasticity. Early in development spontaneous vesicle exocytosis (SVE), often considered as 'synaptic noise', is important for homogenization of synaptic weights and maintenance of synaptic weights in the appropriate dynamic range. Our results demonstrate that SVE has a permissive, whereas subsequent evoked vesicle exocytosis (EVE) has an instructive role in the expression of Hebbian plasticity. A timed onset for Hebbian plasticity can be achieved by switching from SVE to EVE and the balance between SVE and EVE can control the effective rate of Hebbian plasticity. We further show that this developmental switch in neurotransmitter release mode enables maturation of spike-timing dependent plasticity. A mis-timed or inadequate SVE to EVE switch may lead to malformation of brain networks thereby contributing to the etiology of neurodevelopmental disorders.

## Author Summary

Neurotransmitter release is the principal form of chemical communication in the brain. When an action potential reaches a synapse, calcium influx activates the machinery for neurotransmitter release. During early neuronal development this machinery matures such that neurotransmitter release becomes time-locked to action potentials. By modeling this change in neurotransmitter release, we mechanistically show that the maturation process can be solely responsible for switching on associative (i.e. Hebbian) plasticity in the brain. The relevant proteins of the release machinery can thereby regulate the rate at which neural circuits represent sensory input, providing a novel mechanism to control the learning rate and onset. Appropriately timing of the onset of Hebbian plasticity is important because during early development sensory experience fine-tunes, often irreversibly, the neural wiring in our brain.

## Introduction

Functional circuits in the brain are rapidly established during early development and are fine-tuned by experience throughout life. In the rodent neocortex, for example, cortical columns form in the first three weeks after birth [1,2]. During this period, thalamo-cortical input is essential for columnar formation [3] and stimulus-evoked activity patterns further refine cortical connectivity [4]. Activity-dependent forms of synaptic plasticity, in particular Hebbian plasticity, guide the cortical refinement and are required for functional maturation of cortical circuits [5]. Although the postsynaptic pathways involved in the developmental stages of synaptic plasticity are well characterized (e.g. [6,7]), the cellular mechanism on the presynaptic side that triggers the onset of Hebbian plasticity is still unclear [7].

Early in development, pre- and postsynaptic structures are co-localized, even in the absence of any action potential activity [8], while functional connectivity emerges later in development [8]. Initially, synapses are thus established, but functional communication between neurons is lacking. During this initial phase, spontaneous vesicle exocytosis can help to maintain synapses [9,10]. Reduced vesicle release during development reduces the rate of synapse formation (for review see [11]). Here we studied the role of vesicle exocytosis for maturation of associative plasticity. We propose that a switch in vesicular exocytosis mode ensures a discrete onset for Hebbian plasticity and triggers the activity-dependent neural circuit formation during neurodevelopment.

**Fig 1:**
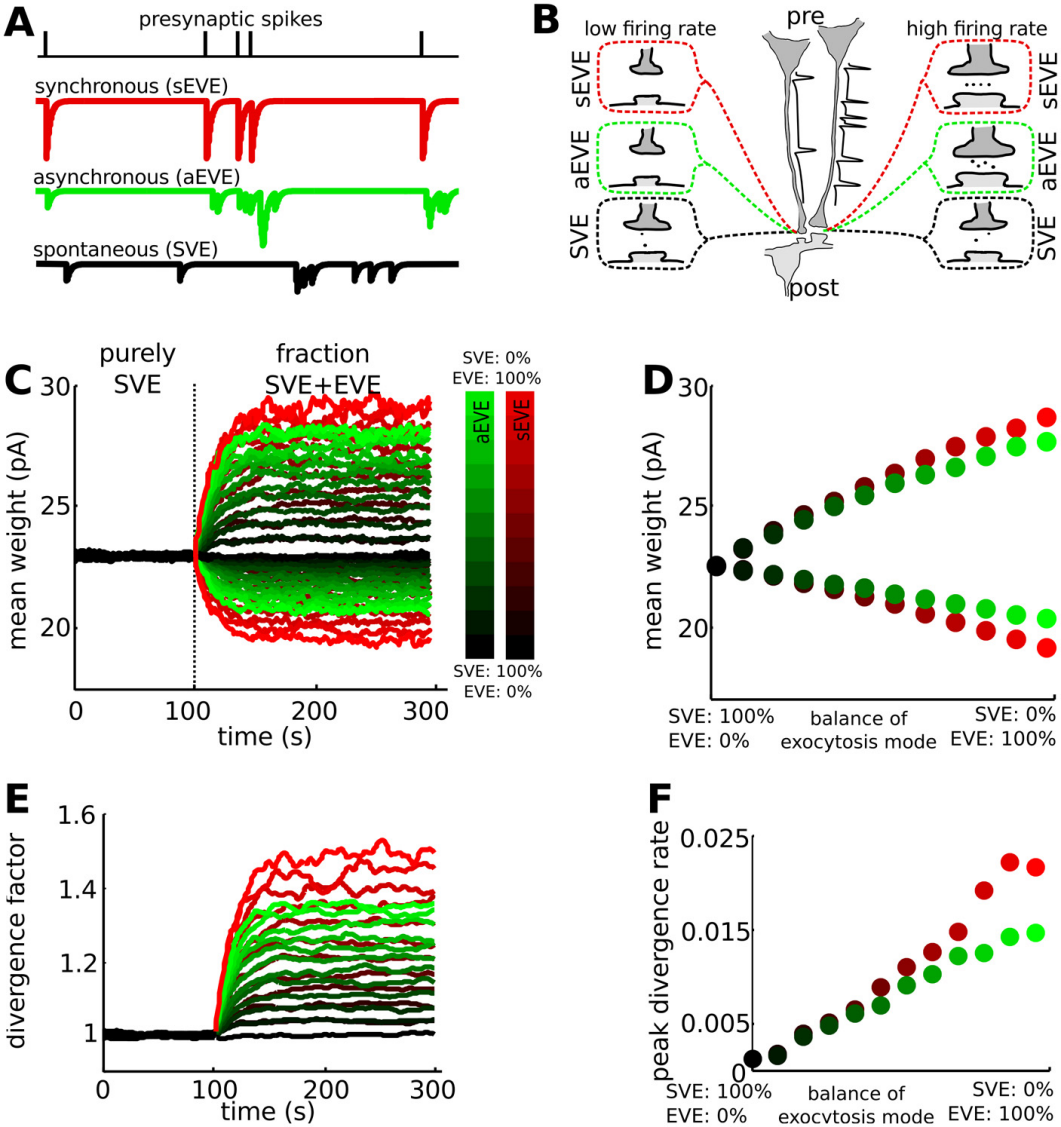
The balance between spontaneous and evoked vesicle exocytosis provides control for the rate of Hebbian plasticity. We use the VTDP model represented by Eqs (1) to (8). **A**: Vesicle exocytosis can occur directly following a presynaptic spike (sEVE), with a high probability after a presynaptic spike (aEVE) or randomly, independent of a presynaptic spike (SVE). **B**: Each exocytosis mode can coordinate activity across the synapse. When EVE mode dominates the release process, there will be competition between synapses of neurons that have low (left presynaptic neuron) and high (right presynaptic neuron) firing rates, with the former decreasing in strength and the latter increasing in strength (top and middle synapses). There is no such competition for SVE dominated synapses (bottom synapses). **C**: Vesicle exocytosis is initially dominated by SVE, which maintains the synaptic strength distribution. Following a switch to EVE, the synapses at which many action potentials arrive (upper traces) are potentiated at the expense of synapses for the lower firing rate neurons (lower traces). The (partial) switch to EVE occurs at the dotted line and is either to aEVE or sEVE. The colorbar denotes the SVE-aEVE (green) and SVE-sEVE (red) balance. **D**: The final difference in weight for the synapses of the low- and high firing rate neurons depends on the SVE-EVE balance. Dots are the mean of the last 100 seconds for the upper (top) and lower (bottom) traces in panel C. Colors represent the same degree of balance as in panel C. **E**: The divergence factor between the synapses of the high and low firing rate neurons (upper and lower traces in panel C, respectively) increases after the SVE to EVE switch. **F**: The divergence rate is calculated as the change in divergence factor (panel E) per time unit. The maximal rate of divergence depends on the SVE-EVE balance, where a large fraction of EVE results in faster divergence. The divergence rate for equivalent strength is a bit lower for aEVE compared to sEVE. For purely SVE there is no divergence. The SVE-EVE balance can thus act to control the divergence rate during synaptic competition.

The mode of vesicular exocytosis changes rapidly during early development [12]. In immature synapses, neurotransmitter vesicles spontaneously fuse with the membrane whether or not there was a preceding action potential (spontaneous vesicle exocytosis, SVE), whereas in mature synapses evoked vesicle exocytosis (EVE) dominates synaptic communication [12]. EVE can occur within an interval of several milliseconds (synchronously evoked vesicle exocytosis (sEVE)) or delayed up to several hundred milliseconds (asynchronous evoked vesicle exocytosis (aEVE)) after the action potential (Fig 1A). Each mode of vesicle exocytosis has the ability to coordinate activity across a synapse, potentially leading to associative plasticity in neural networks. Immature neurons, due to their high input resistance, small size, slow membrane time constant and prolonged decay constant of the excitatory postsynaptic potential (EPSP) [13,14], have a relatively high probability to generate an action potential resulting from postsynaptic integration of uncorrelated synaptic inputs (SVE). The impact of vesicle exocytosis on synaptic communication changes with development. With reduced input resistance and a faster decay of EPSP [13,14] the postsynaptic window of opportunity for coincidence detection is shortened such that only temporally correlated presynaptic activity (EVE) can be efficiently integrated by the postsynaptic neuron.

While the molecular mechanisms for the three principal modes of vesicle exocytosis (SVE, aEVE, sEVE) have been characterized [15], the functional role for SVE and aEVE is currently not clear [15–17]. Here, by modeling the developmental changes in exocytosis mode, we show that SVE optimally prepares neural circuits for learning by maintaining synaptic weights in the appropriate dynamic range and homogenizing the synaptic weights into a tight homogeneous distribution. A switch to EVE initiates a period of rapid synaptic plasticity where synaptic input can be efficiently stored in the network. The SVE-EVE balance regulates the rate of Hebbian plasticity in the synapse and learning in neuronal networks. Besides the role of asynchronous exocytosis in gain modulation [18], we show that increased locking of vesicle exocytosis to the action potential causes maturation of the STDP rule [19]. These results argue that SVE, often considered as 'synaptic noise', plays an important role in synaptic plasticity during neurodevelopment and predict that a developmentally regulated switch in the mode of vesicular exocytosis contributes to the activity dependent organization of the developing circuits.

## Results

Effective communication across a chemical synapse starts with vesicular exocytosis and subsequent diffusion of neurotransmitters to the postsynaptic terminal. We studied the effect of the three primary modes of vesicular exocytosis (SVE, aEVE and sEVE) on synaptic competition between synapses using a vesicle-timing dependent plasticity model, where presynaptic neurotransmitter release correlated with postsynaptic activity was necessary for Hebbian plasticity. We subsequently used a rate model to characterize the differential effects of the vesicle exocytosis modes on the ability of a neural network to represent a particular pattern of action potentials in matching synaptic weights.

### Vesicle-timing dependent plasticity (VTDP)–modeling framework

Most computational studies of synaptic plasticity with spiking neurons use a spike-timing dependent plasticity (STDP) rule [20,21], implicitly assuming that a presynaptic action potential leads to correlated vesicle exocytosis within a short interval. We focus on the timing of vesicular exocytosis, irrespective of whether it is preceded by presynaptic action potentials, leading to vesicle-timing dependent plasticity (VTDP model). Vesicles that are exocytosed within a canonical STDP time window contribute to time-dependent weight changes as described by the STDP model (Eq (7)).

Vesicle exocytosis can occur in three different modes (Fig 1A and 1B). For SVE, exocytosis is independent of the arrival of an action potential at the presynaptic terminal [15]. For aEVE, exocytosis depends on the calcium concentration and the timing of exocytosis is therefore only loosely coupled to an action potential; each action potential induces a presynaptic calcium influx, whose intracellular concentration exponentially decays. For sEVE, exocytosis immediately follows an action potential.

To study synaptic competition using the VTDP model, we simulated a network model comprised of feed-forward connections from 500 presynaptic neurons to 10 postsynaptic neurons. A subset of the presynaptic neurons (20%) had a higher firing rate (8 Hz) compared to the rest (4 Hz). We used a Poisson process to generate action potentials at these rates. In turn, vesicle exocytosis from the presynaptic terminal was modeled as Poisson processes for SVE, aEVE and sEVE. In the model the probability of exocytosis depended on proteins that are available to support each of the different exocytosis modes. The switch from SVE to EVE is implemented by reducing the fraction of vesicles that are available for SVE and increasing the fraction of vesicles that are available for aEVE or sEVE such that the sum of the fractions remains constant. The probability for vesicle exocytosis also depended on the fraction of available vesicles at the synapse (Eq (3)).

The rate of vesicle release for sEVE was directly related to the firing rate of each neuron. The release rate of SVE was scaled with the mean firing rate of all presynaptic neurons to obtain the same mean release rate as EVE. The rate of asynchronous release depended on the amount of residual Ca^2+^, which decays exponentially (Eq (4)). The total amount of Ca^2+^ entering the presynaptic terminal in response to a single action potential was also scaled to yield the same mean release rate as the other exocytosis modes. Thus, to effectively titrate the differential contribution of the release rate to synaptic plasticity, the cumulative release was normalized such that the mean and total vesicle release rates were equal for the three modes of neurotransmission.

An exocytosed vesicle was moved from the active pool to the recycling pool. Recovery from the recycling pool to the active pool was modeled as an exponential process (Eq (5), [22]). The neurotransmitters released from each vesicle induced an excitatory postsynaptic current that decayed within several ms. We used leaky integrate-and-fire neurons with an adaptive spike threshold for the postsynaptic neurons. A homeostatic rule kept the firing rate of the postsynaptic neurons at the desired dynamic range (~5 Hz) by scaling the synaptic weights accordingly (Eq (8)).

Thus, for SVE, vesicle exocytosis was spatially (across synapses belonging to the same neuron) and temporally (across time within individual synapses) random and was not coupled to the onset of presynaptic action potential (Fig 1B). For aEVE, vesicle exocytosis was spatially biased to synapses belonging to presynaptic neurons with higher firing rates, but it was temporally diffuse (Fig 1B). For sEVE the vesicle exocytosis was both temporally and spatially linked to the spiking activity of the neurons (Fig 1B).

### Spontaneous exocytosis has a permissive and evoked exocytosis has an instructive role in Hebbian plasticity

Synaptic plasticity leads to competition between synapses when presynaptic cells are active at different rates [20]. In the VTDP model, synapses that were active prior to a postsynaptic action potential were strengthened whereas the others were weakened. We used the VTDP model to study how the three vesicle exocytosis modes contribute to synaptic competition. When synaptic communication was mediated by SVE alone, the chemical neurotransmission did not lead to competition between synapses onto the same postsynaptic neuron, and maintained synaptic weight distribution to a homogeneous distribution, also referred to as a unimodal distribution (Fig 1C). Introducing a (partial) switch to either aEVE or sEVE caused divergence between synaptic weights for the synapses of the high and low firing rate neurons. The gradual change in synaptic efficacy results in a heterogeneous synaptic efficacy distribution in the network, with synapses that were either weak or strong, but fewer of the intermediate strength (referred to as multimodal). The amount of divergence (the degree of separation between high and low synaptic weights) between synapses of the low- and high firing rate neurons depended on the SVE-EVE balance (Fig 1D). When vesicle exocytosis was dominated by EVE we found that asynchronous exocytosis resulted in a moderate, and synchronous exocytosis resulted in a strong drive to increase the synaptic weights of the higher firing presynaptic neurons at the expense of synapses of the lower firing rate neurons (Fig 1E). The SVE-EVE balance regulated the rate at which synaptic competition occurred (Fig 1F). Correlated input could thus shape the structure of the network via EVE. As the rates of vesicle exocytosis onto the postsynaptic neuron were normalized such that they are equal for the three modes, this difference in outcome is caused by their spatiotemporal structure of the synaptic inputs to the postsynaptic neuron. This model of vesicle-timing dependent plasticity shows that the SVE-EVE balance can regulate the rate with which new patterns of action potentials are imprinted into synaptic weights. A switch from SVE to EVE enables a discrete onset for Hebbian plasticity and initiates a period at which synaptic weights rapidly rewire to represent dominant sensory activity. The SVE-EVE balance can control the rate of Hebbian plasticity.

### Additional functional roles for SVE

**Fig 2:**
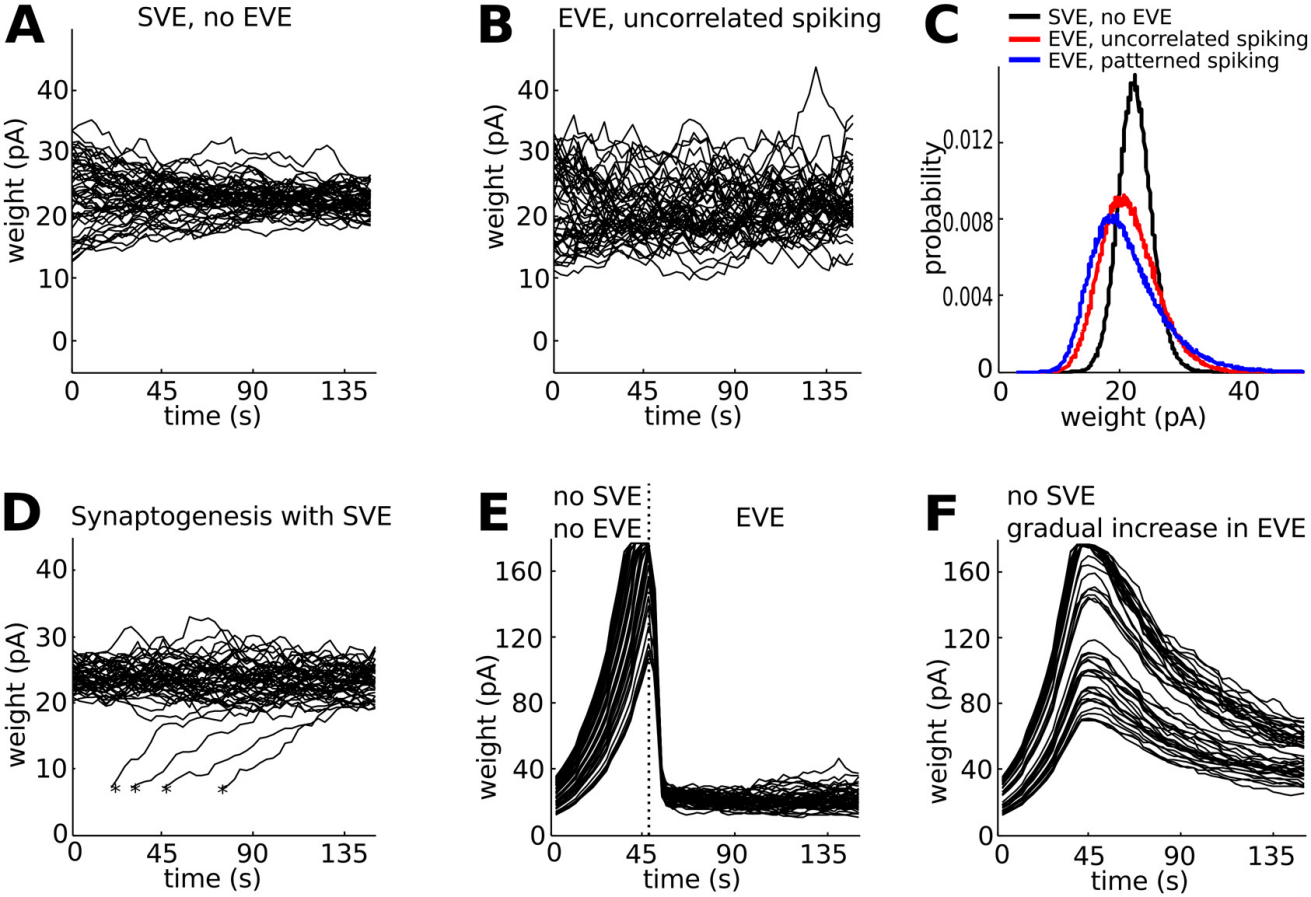
Additional functional roles of spontaneous vesicle exocytosis (SVE). **A**: Synaptic weights that are initially heterogeneous, homogenize into a tight synaptic weight distribution in the case of SVE. Black lines are traces of 50 synaptic weights randomly selected from a network of 500 presynaptic neurons that connect to 10 postsynaptic neurons. The neurons fire uncorrelated action potentials at the same mean firing rate. **B**: Synaptic weights show large fluctuations in the case of EVE. As before, neurons fire uncorrelated action potentials at the same mean firing rate. **C**: Distribution of synaptic weights for SVE (black line), EVE with uncorrelated spiking activity (4.8 Hz, blue line) and EVE with patterned spiking activity (red line). For the patterned, a subset of the neurons (20%) have higher firing rates (8 Hz) compared to the others (4 Hz). Notice the broadening for uncorrelated and patterned spiking activity in the case of EVE. **D**: Synaptogenesis creates new synapses with small initial synaptic weights (denoted with a *). SVE incorporates the newly generated synapses into the homogeneous pool of all synaptic weights. **E**: In the absence of SVE, the synaptic weights increase due to the homeostatic mechanism until they reach the max synapse size. Synaptic weights are heterogeneous when the switch to evoked vesicle exocytosis (EVE) occurs. **F**: For a gradual increase of EVE, in the absence of SVE, the synaptic weights span a large dynamic range and are heterogeneous distributed.

In addition to the above described functional role for SVE to control the rate of Hebbian plasticity, we explored other functional roles for SVE. We first compared the effect of SVE (Fig 2A) to that of EVE with uncorrelated spiking activity (Fig 2B) on the distribution of synaptic weights. We found that SVE homogenizes the synaptic weights into a tighter weight distribution compared to EVE (Fig 2C). SVE events occur more diffusely compared to EVE, and furthermore the larger EPSC inputs from EVE result in stronger correlations between pre- and postsynaptic spiking. Thus, an additional functional role of SVE is homogenization into a tight synaptic weight distribution and increased stability against synaptic weight fluctuations compared to EVE. Homogenization is also important for synaptogenesis, because newly formed synapses with small initial weights grow stronger and become incorporated into the synaptic pool (Fig 2D).

Next we asked whether SVE during early development was necessary at all; and what would happen for a gradual increase in EVE in the absence of SVE. Synaptic fluctuations as well as divergence in synaptic weights due to differences in firing rate can be prevented by completely abolishing vesicle release (no SVE, no EVE). If neither SVE nor EVE are present, synaptic weights become saturated due to the homeostatic mechanism (Fig 2E, Eq (8)). If EVE increases gradually during development, synaptic weights also increase due to the homeostatic mechanism, while remaining heterogeneously distributed (Fig 2F). Thus SVE is necessary to maintain synaptic weights in the appropriate dynamic range. SVE and EVE release mechanisms thereby both serve a biological role in synapse formation and synaptic maintenance [9–11].

### Rewiring an established neuronal circuit after the SVE to EVE switch requires prolonged synaptic communication

**Fig 3:**
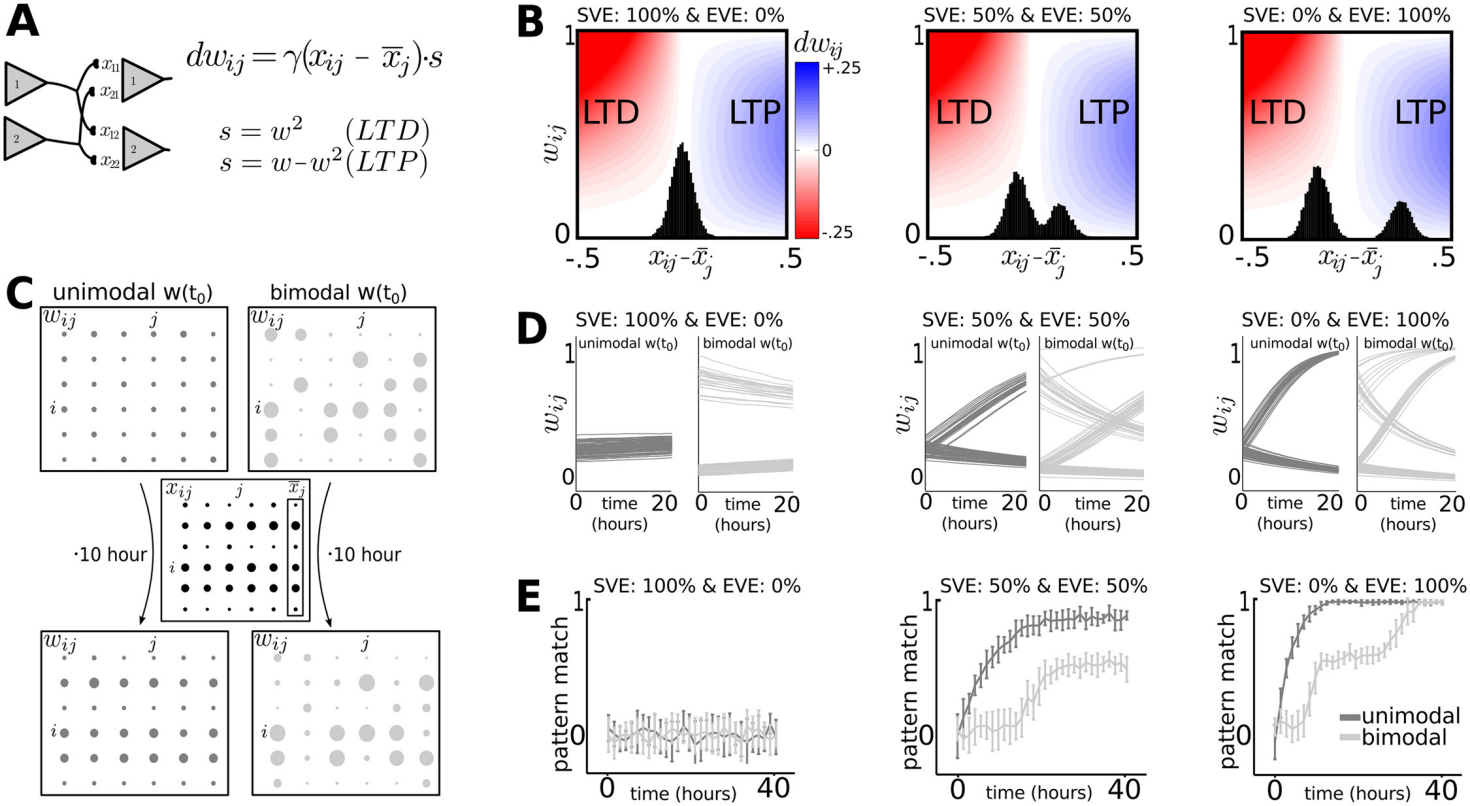
The rate at which a pattern of action potentials is imprinted onto a configuration of synaptic weights depends on the initial weight distribution and the SVE-EVE balance. We used the rate model described by Eqs (11) and (12). **A**: Presynaptic neurons *i* connect to postsynaptic neurons *j*, for which the synaptic weights are shaped by local competition between the vesicle exocytosis rates (x*ij* relative to the average exocytosis rate onto the postsynaptic neuron (${\bar{x}}_{ij}$)). The exocytosis rates are a combination of SVE (randomly distributed across synapses) and EVE (strong for the subset of highly active presynaptic neurons). **B**: The degree of potentiation (blue, positive rate of change in *dw_ij_*) and depression (red, negative rate of change) depends on whether the relative exocytosis rate is bimodal versus unimodal (black histograms). **C**: The initial synaptic weights (*w*) have either a unimodal distribution (left) or a bimodal distribution (right). A pattern of activity (*x*) is presented to both networks. Using the rate model, we can clearly see the activity pattern is represented in the weights of the network that started with the unimodal distribution, but not for the initial bimodal distribution. Dot size represents weight (*w*_ij_) or exocytosis rate (*x*_ij_). **D**: The rate at which synaptic weights converge to a configuration corresponding to the applied patterns of action potentials depends on the SVE-EVE balance. Dark and light gray traces start from an initial unimodal and bimodal distribution respectively. **E**: Matching the synaptic weights to the pattern of action-potential activity (pattern match, Eq (13)) takes significantly longer when the initial weights have a bimodal distribution.

After the switch from SVE to EVE and subsequent changes in synaptic weight distribution across synapses occurred, the network now consists of a few strong synapses among many other weak ones [20,23]. This change in synaptic weight distribution shapes the network’s ability to encode patterns of action potentials and translate them into memory traces. We aimed to establish the differential roles of the exocytosis modes in the activity dependent organization of cortical circuits. The VTDP model implements the plasticity terms and vesicle exocytosis times separately for each synapse. Such operations are on matrices of size N_i_ x N_o_ (number of input neurons x number of output neurons), which is computationally more demanding than the classical STDP modeling which generates spike times and thus works with operations on vectors of size N_i_ and N_o_. We therefore used a rate model that represents vesicle-timing dependent plasticity, consisting of a similar structure and that can be implemented with better computational efficiency (Fig 3A). The rate model will help other computational studies that want to conduct follow-up studies to implement our model efficiently. The exocytosis rates are a combination of SVE (randomly distributed across synapses) and EVE (strong for the subset of highly active presynaptic neurons). The evolution of synaptic weights is then followed over time. Synaptic efficacy is potentiated for synapses with high vesicle exocytosis rates at the expense of synapses that have lower rate of exocytosis (Fig 3B).

Each network was initialized either without any particular structure, with each synaptic strength set randomly according to a unimodal distribution, or with a previously encoded weight pattern, comprised of a bimodal distribution of low and high values, that is different from the pattern presented (Fig 3C). While no pattern of action potentials could be stored during SVE, synaptic communication through EVE introduces competition that enables storage of the patterns of action potentials (Fig 3D). A switch from SVE to EVE thus generates a window of opportunity for a cortical network to rewire as instructed by experience. Rewiring an established circuit after the switch takes significantly more time than starting from a homogeneous state without patterns of action potentials imprinted (Fig 3E).

SVE can thus be used to maintain baseline network activity and unspecific network connectivity, and the SVE-EVE balance regulates the rate at which time neural circuits are shaped by patterns of action potentials.

### The developmental switch from SVE to EVE during human brain development

How is the SVE-EVE balance regulated? An initial suggestion that SVE and EVE might utilize distinct vesicle pools [24] was accompanied by a concerted research effort to characterize the differential regulatory pathways that impact vesicle exocytosis (for reviews, see [15,25]). Optical recordings from developing neurons *in vitro* demonstrated a switch from SVE to predominantly EVE as neurons matured [12]. The switch to EVE at the synaptic terminal occurs independent from whether there is functional communication with a postsynaptic neuron, suggesting a cell-autonomous process [12]. Investigations in cell-autonomous regulation of vesicle exocytosis focus on the functional properties of the proteins involved in the docking of vesicles to the presynaptic membrane such as the SNARE proteins Syntaxin, SNAP-25, and VAMP2/Synaptobrevin and a core set of SNARE-binding proteins: Synaptotagmin, Munc13, Munc18, Doc2 and Complexin [25].

**Fig 4:**
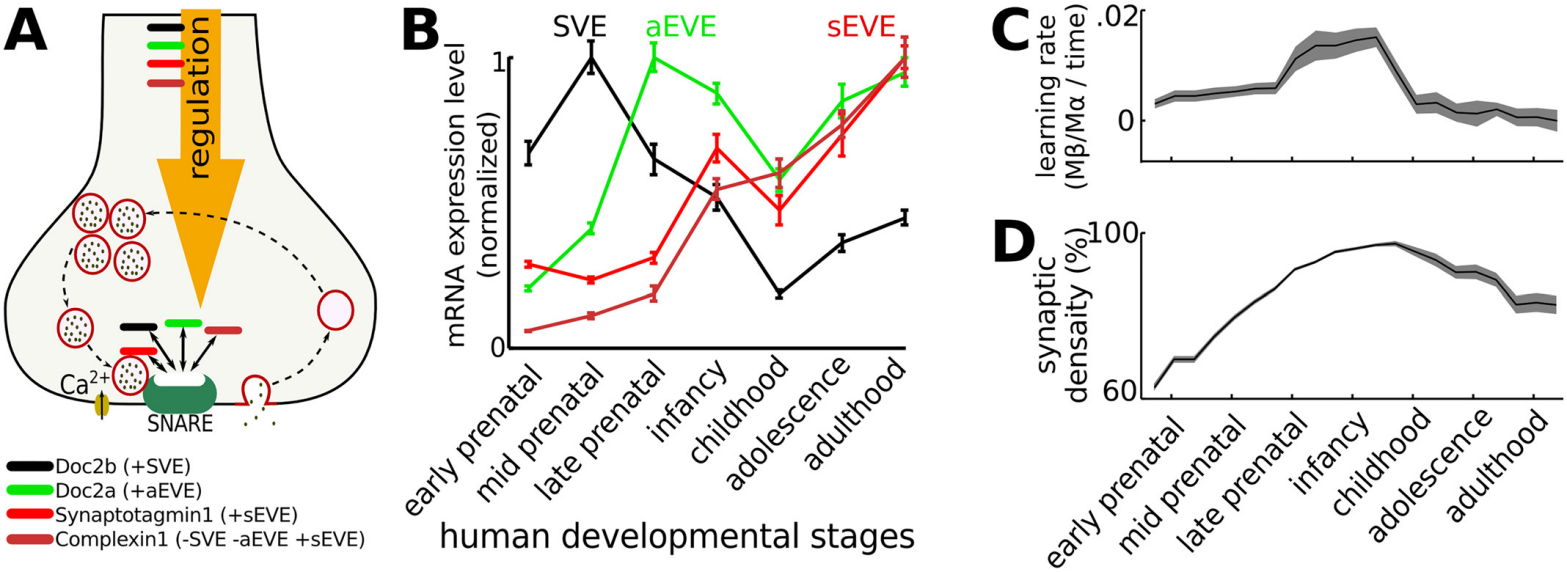
During cortical development there is a switch from exocytosis dominated by spontaneous exocytosis to asynchronous and synchronous vesicle exocytosis. **A**: The SVE-EVE balance is regulated by Ca^2+^ sensing proteins (Doc2s and synaptotagmins). Complexin1 promotes Synaptotagmin1 mediated synchronous exocytosis while blocking Spontaneous and asynchronous exocytosis [29]. **B**: In humans, the relative mRNA expression of these sensors varies in time. Early in development there are high levels of Doc2b (SVE, black line), which decay during the intermediate developmental stage at which time there is an increase in Doc2a (aEVE, green line) expression. The expression of Synaptotagmin1/Complexin1 (sEVE, red lines) increases throughout neurodevelopment, reaching their maximal expression levels during late development [31]. **C**: By implementing the mRNA expression levels as ratios for SVE, aEVE and sEVE, a period of rapid learning is observed early in development during which SVE decreases and aEVE and sEVE increase. During this period the wiring in the neural circuitry is formed, after which the learning rate decreases. Gray shade is standard deviation. **D**: During early developmental periods synaptogenesis is prominent, until activity-dependent pruning reduces the synaptic density later in life.

Exocytosis of vesicles docked at the active zone in the presynaptic terminal is mediated by calcium-sensitive proteins. Calcium sensitivity and kinetics of vesicle exocytosis depends on the calcium sensors expressed. The presence of high-affinity calcium sensor Doc2b increases the fraction of SVE exocytosis [26], which might also act completely independent of presynaptic action potential induced calcium entry [27]. Doc2a on the other hand facilitates aEVE [28]. sEVE is mediated by Synaptotagmin1, which is selectively enhanced by Complexin that can act as a fusion clamp or adaptor to inhibit SVE and aEVE [29,30] (Fig 4A).

The expression of these calcium sensors varies across time, which is reflected in the corresponding mRNA expression levels. We re-analyzed the human transcriptome [31] for mRNA expression profiles of the calcium sensitive proteins during neurodevelopment and found high Doc2b expression early in development was followed by a peak in Doc2a, while Synaptotagmin1/Complexin1 became prominent during early childhood (Fig 4B). These profiles are consistent with a switch from high SVE to aEVE early in development and dominant sEVE at later developmental stages. To study the developmental learning rate, the SVE, aEVE and sEVE ratio profiles were used to mimic a biological situation. The synapses belonging to the higher firing rate neurons strengthen at the expense of the synapses belonging to the lower firing rate neurons (for EVE, not for SVE). The learning rate (see Eq (9) in the Materials & Methods) diminishes if the synaptic weights approach their upper and lower bounds, which resulted in a learning rate peak around infancy (Fig 4C). During development, new synapses were formed [32] and weak synapses were pruned ([33], see Materials & Methods). Early in development the rate of synaptogenesis was larger than the rate of synaptic pruning, which caused a rise in synaptic density (Fig 4D). After EVE became more dominant, the rate of synaptic pruning became larger than synaptogenesis, which caused a decrease in synaptic density.

The SVE-EVE switch thus coincides in time with the early stages in human neurodevelopment [34,35]. There is a rapid increase in synaptogenesis around the time of birth for all cortical areas, while a burst of synapse formation occurs at different ages in cortical regions [36]. In the visual cortex, for example, synapse formation is accelerated between 3 and 4 months and the maximum density is reached between 4 and 12 months, while synaptogenesis in the prefrontal cortex reaches its peak well after the first year [34]. After the peak in synaptic density, corresponding to about 150% of adult levels, a sensitive period with high levels of associative plasticity occurs, whereas plasticity that occurs beyond the end of this sensitive period alters connectivity patterns within the architectural constraints established during the sensitive period [34,35]. These developmental patterns are highly conserved throughout many species [36].

### The maturation of the STDP rule during development

**Fig 5:**
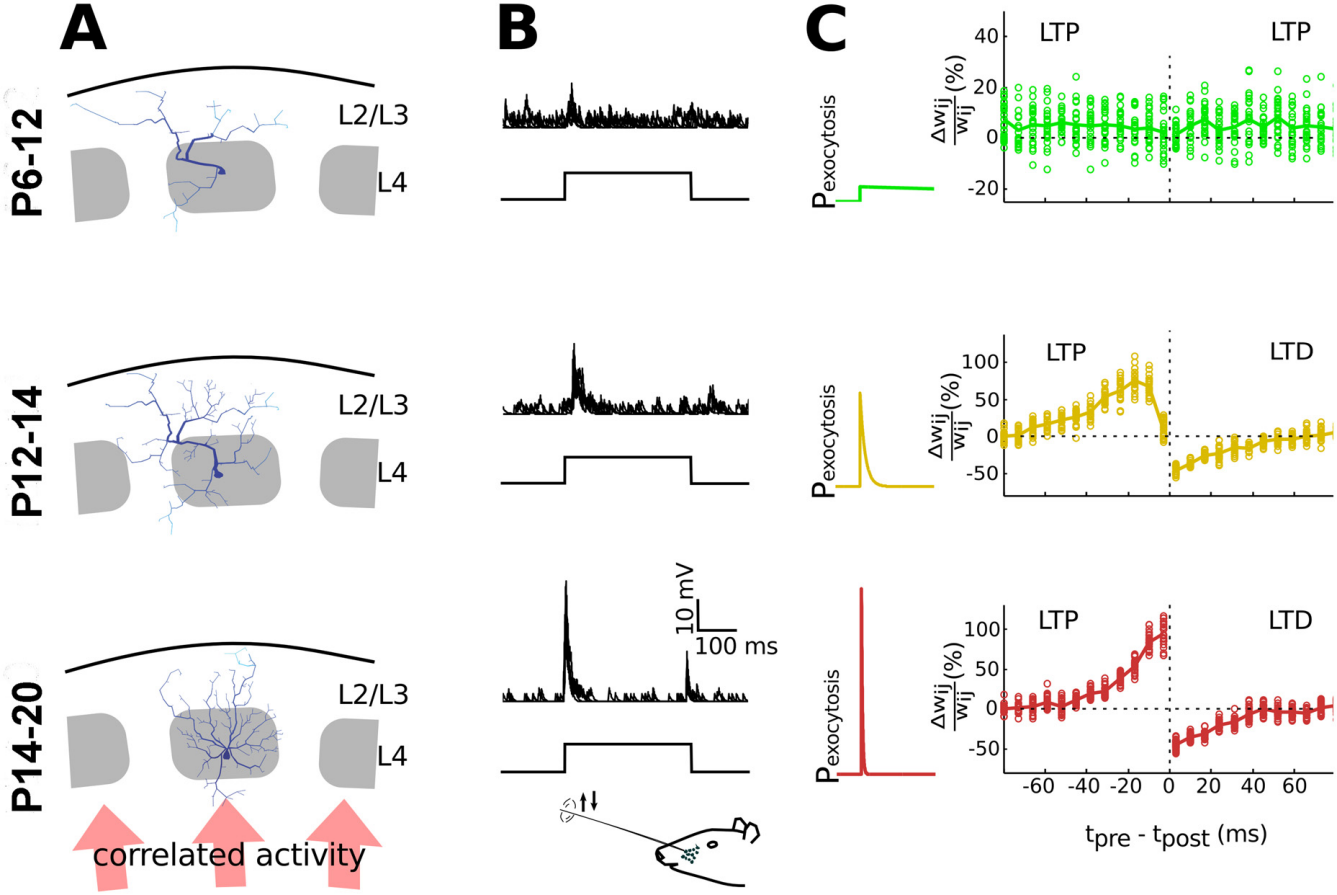
Predictions for the role of the SVE to EVE switch in refinement of cortical neural circuits. **A**: During early development arborization of barrel cortical neurons is sparse, and does not respect the columnar boundaries. Neurite outgrowth occurs during the intermediate developmental period and in late development, as synapses mature, the extensive arborizations outside the column are pruned in an activity dependent manner (illustration based on the results from [1], using the Trees Toolbox [37]). **B**: Whisker deflection induces synaptic responses in layer 2/3 of the barrel cortex. The input to L2/3 is largely uncorrelated at P12, with a rapid switch to stimulus-driven responses during development [2]. At P12-P14 the stimulus-induced activity is typically prolonged and with high variability, whereas at a later stage (P20) the responses are more precisely time-locked to the stimulus [2]. **C**: By modeling changes in the release probability from SVE to highly time-locked release during development, Hebbian plasticity behavior changes from an immature to mature STDP rule. The maturation process of the STDP rule is described *in vitro* for layer 4 –layer 2/3 synapses during these critical developmental stages [19]. The calcium time constant (Eq (4)) was 1 second (top), 7 millisecond (middle) and 2 millisecond (bottom), and 50 stimuli were given for each t_pre_ - t_post_ bin.

A transition from an immature to mature STDP rule is observed during early cortical development of the rat somatosensory cortex (Fig 5C, [19]). The transition coincides in time with a critical period in circuit formation in the barrel cortex, during which extensive activity-dependent pruning occurs, confining neuronal arborizations to the columnar structure in the barrel cortex ([1], Fig 5A). At the same time, a functional transition occurs from predominantly spontaneous, stimulus-indepedent activity early in development to stimulus-dependent activity later in development ([2], Fig 5B). The action potential-dependent responses in the intermediate stages are typically prolonged and with high variability, which matures to responsiveness that is tightly synchronized to the stimulus. By modeling the corresponding changes in exocytosis using the VTDP model (see above), we found that STDP expression matures because of the switch to vesicle exocytosis that is increasingly time-locked to the stimulus ([19], Fig 5C).

## Discussion

The switch from SVE to EVE triggers a period of rapid synaptic competition, mediated by synaptic depression and potentiation according to Hebbian plasticity rules. This may seem trivial, but it has important functional consequences for neural circuit development, as we will highlight here. In general, the switch provides a framework for understanding the onset of synaptic plasticity in circuit formation during neurodevelopment by mechanistically linking the onset to the developmental changes in vesicular exocytosis of neurotransmitters.

While the molecular mechanisms for vesicle exocytosis are well described, the functional roles of SVE and aEVE are unclear [15–17]. Our VTDP model showed that early in development, in the absence of relevant sensory input, SVE maintains synaptic weights in a homogeneous distribution and within the appropriate dynamic range. Molecular changes at the presynaptic terminal can then switch vesicle release from SVE to EVE. We propose that this SVE to EVE switch enables sensory inputs to fine-tune the circuit connectivity using activity dependent synaptic reorganization (Fig 5, [38]). The transition from SVE to EVE can initiate a window of strong associative plasticity, during which for example local connectivity can be reorganized extensively by activity-dependent mechanisms of plasticity such that the appropriate feature selectivity is established in the network [5]. Similar to the switch from immature (P9-11 in mice) to mature (P12+) visual responses, a switch from endogenous network oscillations to mature visual responses occurs at birth in human [39]. Children born prematurely have deficits in visual acuity and contrast sensitivity compared to children born at full term [40].

The SVE to EVE switch involves molecular changes at the presynaptic terminal and brain regions could open windows for rapid synaptic reorganization in a specifically programmed temporal order. Hierarchical organization of sensory-motor pathways may develop through a cascade of such windows for plasticity [41]. The sequential opening of such windows was for example observed for the visual cortex, where plasticity for inputs to L2/3 extends beyond the window of plasticity for thalamic inputs to L4 [42].

For an understanding of circuit plasticity during development, one needs to take into account the synaptic learning rule, the spike activity patterns present, and the timescales of their information content [43]. For example, the timescales at which STDP operates can change the effectiveness of synapse refinement, and specifically STDP over short timescales does not necessarily lead to the appropriate circuit refinement during early development [44]. During development correlations in spike activity patterns become faster, which can further change the efficacy of STDP [43]. Importantly, the developmental switch that we found was observable for different timescales of STDP and thus timescale independent.

Burst-based Hebbian learning rules, which integrate spike activity patterns over longer timescales, play a role in cortical refinement [45]. For burst-based plasticity, LTP is favored over LTD [46]. Vesicle exocytosis in the form of aEVE (vesicles are released asynchronously after an action potential) could be considered a miniburst of vesicle exocytosis. Such a miniburst of vesicles introduces multiple pairings of vesicles with a postsynaptic spike as opposed to sEVE (vesicles are released directly after an action potential and have the same temporal relation to the postsynaptic spike). The release in the form of aEVE could thus bias the VTDP rule towards LTP over LTD early in development [46].

Neural responses become increasingly time-locked to stimuli during development [2,39]. Using the VTDP model, we show that the transition to action potential-dependent release can explain the maturation of the STDP rule observed in the somatosensory cortex ([19], Fig 5C). The VTDP model thus links together the experimental observation of increased action potential-dependent input, receptive field refinement to the home column of sensory input and maturation of STDP that coincide in time during early postnatal development. Although plasticity mechanisms in the postsynaptic neuron change during development [47], our model does not depend on the molecular machinery for plasticity in the postsynaptic neuron. Solely by introducing changes in the presynaptic release machinery, our VTDP model can mechanistically elucidate the functional changes, structural reorganization and maturation of activity-dependent plasticity that occur during early neurodevelopment.

The early postnatal development is accompanied by periods with rapid, often irreversible changes [48], including but not limited to maturation of inhibition [49,50]. A modeling study shows that the maturation of inhibition can initiate a critical period by preferentially suppressing spontaneous spiking activity relative to visually evoked activity [41]. For this mechanism to be effective it should occur after the SVE-EVE switch, otherwise no evoked activity could be preferentially selected. Furthermore, the maturation of inhibition effectively increases the spiking threshold which affects all synapses simultaneously, whereas a SVE-EVE switch could initiate competition between synapses based on the identity of the presynaptic populations, thereby regulating synaptic plasticity in a region, layer or neuron type specific manner. The balance between SVE and EVE was shown to segregate between synapses, allowing the SVE-EVE switch to occur in a synapse specific manner [51,52]. The increase in inhibition could further functionalize the SVE-EVE switch by the selective suppression of SVE relative to EVE [41]. However, the increased threshold caused by the maturation of inhibition could be compensated by homeostatic upscaling of all synapses, potentially abolishing the effect of inhibition.

The results presented herein shows that SVE independently and single-handedly places synaptic rewiring on hold, and switching to EVE induces a period of rapid, activity-dependent changes. Once the neural circuit is wired to represent particular sensory features, rewiring is significantly more difficult and requires disproportionately more vesicular neurotransmission.

### The cell-autonomous regulation of the SVE-EVE balance

At the presynaptic terminal, proteins involved in the exocytotic machinery determine the SVE-EVE balance. The expression of the relevant calcium sensitive proteins can be genetically regulated, and evidence for a cell-autonomous switch from SVE to EVE has previously been shown *in vitro* [8,12].

Despite the fact that both SVE and EVE have the potential to drive Hebbian plasticity across the synapse, it is unclear how, where and when Hebbian plasticity develops in the first place [7]. The model presented here mechanistically links the mode of vesicular exocytosis to Hebbian plasticity during neural development. As the switch from SVE to EVE is genetically regulated, it is an ideal candidate mechanism to initiate region, layer and cell specific onset of Hebbian plasticity. Specific regulation of the onset of Hebbian plasticity is, for example, important for a hierarchical system in order to optimally deal with the input statistics, such that sensitivity to low-level features (i.e. orientation) develop prior to rewiring the circuits that deal with high-level features (i.e. object recognition) [42].

Neural circuits dealing with sensory information first establish diffuse connectivity with broad receptive fields, followed by selection and refinement of the projections (Fig 5A, [1,5]). Whereas electrophysiological recordings of neural circuit development can be biased towards characterizing high firing rate neurons, the role of the SVE-EVE balance is optimally studied with optical and molecular techniques that can assess specific cell activity and collect large population statistics without bias [53]. Further development of molecular techniques to regulate and track the cellular content of the SVE-EVE balance together with optical recording techniques is necessary for fundamental insights in the role of vesicular exocytosis on the structural and functional aspects of synapse maturation and maintenance during neural development.

Our results predict that SVE plays a permissive role in the expression of synaptic plasticity and is important for the maintenance of synaptic strengths by disconnecting the action potential-dependent activity during early neurodevelopment. In agreement with this proposition, selective disturbance of SVE release in *Drosophila* neuromotor junction alters the structural organization of the synapses during development [54].

Understanding the specificity of cell-autonomous regulation of the SVE-EVE balance across time and brain region will provide important insight into the maturation of synaptic wiring and creation of functional networks during brain development. An SVE-EVE imbalance might result in learning deficits because functional circuits would not be appropriately formed by sensory experience in the absence of the regulated switch in the mode of neurotransmitter release. As such, deregulation of the molecular pathways of the three modes of vesicle exocytosis might contribute to the etiology of neurodevelopmental disorders. For example, the expression of Complexin1, a protein that suppresses SVE and enhances EVE, is dysregulated in patients with schizophrenia, depression and bipolar disorder [55]. Complexin1 knock-out mice show developmental deficits [55]. Hence understanding the molecular mechanisms underlying vesicle exocytosis in relation to developmental regulation of Hebbian plasticity may lead to the rational design of treatments that could alleviate learning deficits early in neural development.

## Materials and Methods

To study the role of exocytosis mode on Hebbian plasticity without assumptions regarding structure of the network, we use a feed-forward connection scheme. An input pattern consists of a subset of upstream (presynaptic) excitatory neurons with higher firing rates relative to the rates of the other neurons. The competitive input in vivo can originate from an over-representation of particular sensory inputs or variability in neural excitability [56]. We apply homeostatic plasticity [57] to maintain the firing rates of the postsynaptic neurons within a certain range (~5 Hz), which also ensures competition between the synaptic inputs.

The vesicles can be exocytosed according to three different mechanisms each represented by a fraction, which can vary because it reflects the presynaptic protein content of the relevant Ca^2+^ sensitive proteins for SVE, aEVE and sEVE (Fig 4A, [15,25]). After vesicle exocytosis, the postsynaptic membrane is depolarized, through which each released vesicle can contribute to a time-dependent learning effect. In the case of aEVE and sEVE, the resulting connectivity pattern is comprised of a few strong connections among many weak connections (Figs 1, 2 and Reference [20]).

### Vesicle Timing Dependent Plasticity model

To study Hebbian plasticity in a feed-forward network architecture we use leaky integrate-and-fire neurons:

$$\frac{dV}{dt}=\frac{-(V-V_{rest})}{\tau_{m}}+\frac{I_{ves}}{C_{m}}$$ (1)

The neurons have a resting membrane potential *V*_*rest*_, a membrane time constant *τ*_*m*_ and a capacitance *C*_*m*_ (for parameter values see Table 1).

**Table 1:** Parameters for the VTDP model.

Variable description	Variable name	Value (reference)
Membrane rest potential	V_rest_	-70.6 mV [20]
Membrane time constant	τ_mem_	9.4 ms [20]
Membrane capacitance	C_mem_	281 pF [20]
Spike threshold at rest	θ_rest_	-50.4 mV [20]
Spike threshold max	θ_max_	30.4 mV [20]
Spike threshold relaxation constant	τ_θ_	50 ms [20]
Calcium decay time constant	τ_Ca2+_	100 ms [15]
STDP time constant	τ_STDP_	20 ms [58]
Vesicle recycling rate	τ_rec_	800 ms [22]
Homeostatic plasticity time constant	τ_h_	100 s
Rate constant of rate model	γ	10^−4^ s^-1^
Number of presynaptic neurons	N	500
Firing rate low / high	r_j_	4 / 8 Hz

Each vesicle induces a postsynaptic current I_ves_ (See Eq (6) below). Refractoriness was modeled with the adaptive action potential threshold:

$$\frac{d\theta_{m}}{dt}=\frac{-(\theta_{m}-\theta_{rest})}{\tau_{\theta }}$$ (2)

The adaptive threshold *θ*_*m*_ is reset to *θ*_*max*_ directly after an action potential and returns with a time constant *τ*_*θ*_ to the threshold value at rest *θ*_*rest*_ (for parameter values see Table 1).

We describe vesicle exocytosis in terms of the rate of Poisson processes for SVE, aEVE and sEVE. The resulting probability of exocytosis depends on the number of molecules that are available to support each of the different exocytosis modes, described here in terms of the fraction *ξ*_*SVE*_, *ξ*_*aEVE*_, and *ξ*_*sEVE*_, the sum of which is one per synapse. The switch from SVE to EVE is implemented by reducing *ξ*_*SVE*_ and increasing *ξ*_*aEVE*_ or *ξ*_*sEVE*_, for which ξ_SVE_ = 1- ξ_aEVE_ - ξ_sEVE_. The probability of exocytosis (*v*) during a short interval (we used the Euler Method to solve the differential equations with time steps of Δ*t* = 1 ms), from presynaptic neuron *i* to postsynaptic neuron *j*, depends on the fraction of the available (P_*a*,*ij*_, see Eq (5)) over the total (P_*c*_) vesicle pool:

$$\begin{array}{l} v_{SVE,ij}=n\cdot r_{m}\cdot \xi_{SVE}\frac{P_{a,ij}}{P_{c}}\Delta t\\ v_{aEVE,ij}=Ca_{ij}\cdot \xi_{aEVE}\frac{P_{a,ij}}{P_{c}}\Delta t\\ v_{sEVE,ij}=n\cdot r_{i}\cdot \xi_{sEVE}\frac{P_{a,ij}}{P_{c}}\Delta t \end{array}$$ (3)

For a fair comparison of the different exocytosis modes and have the same average release rate, *v*_*SVE*_ is scaled by the mean firing rate that would drive the number of vesicles released through sEVE (*r*_*m*_ = Σ*r*_*i*_ /*N*) averaged across all *N* presynaptic neurons and *v*_*aEVE*_ depends on the amount of residual Ca^2+^, which decays exponentially with time constant *τ*_*Ca*_ (parameter values in Table 1):

$$\tau_{Ca}\frac{dCa(t)}{dt}=-Ca(t)$$ (4)

The total amount of Ca^2+^ entering the presynaptic terminal in response to a single action potential is given by *A* = *n dt* / *τ*_*Ca*_, such that each action potential is modeled to exocytose 4 vesicles for ξ_aEVE_ = 1. Likewise, the same number (*n* = 4) vesicles is released by sEVE for each action potential. Firing rate is high (8 Hz) for a subpopulation (20%) of the presynaptic neurons compared to the other neurons (4 Hz). The release probabilities are normalized such that for each release mode the average rate of vesicles release is the same, only the spatiotemporal structure of the synaptic inputs onto the postsynaptic neuron is different.

Most synapses in the brain maintain a single active zone [59] with vesicles for docked exocytosis [60], while an axon can make multiple connections between cells. The active zone increases in size during development [61], and the number of docked vesicles is linked to the size of the active zone [62]. We study vesicle pool utilization in the different modes, and set the total vesicle pool per synapse (*P*_*c*_) to 100. A vesicle that is exocytosed, is moved from the active pool (*P*_*a*_) to the recycling pool (*P*_*r*_) and returns to the active pool according to:

$$\tau_{rec}\frac{dP_{a}}{dt}=P_{r}$$ (5)

With *τ*_*rec*_ being the vesicle recycling rate (parameter value in Table 1).

The postsynaptic current (*I*_*ves*_ *(j)*) on neuron *j* is the sum of currents caused by vesicles released from the presynaptic neurons *i* at time *t*_*ves*,*i*_. Each vesicle caused a current *I*_*ves*_ *(i*,*j)* which is described by:

$$I_{ves}(i,j)=w_{ij}\cdot e^{\frac{-(t-t_{ves})}{\tau_{\alpha }}}{\text{ for t}}_{\text{ves}}<\text{t}<{\text{t}}_{\text{ves}}{\text{+t}}_{\text{window}}$$ (6)

With time constant *τ*_*α*_ = 3 ms. For computational efficiency only the currents generated by recently exocytosed vesicles are used, with t_window_ = 20 ms, after which only less than 0.2% of the initial current remains.

Synaptic weights are modified according to the Hebbian learning rules:

$$\begin{array}{l} dw_{\text{ij+}}=\lambda w_{0}^{1-\mu }w_{ij}^{\mu }e^{\frac{-\left|\Delta s\right|}{\tau }}\text{ if}\Delta s>0\\ dw_{\text{ij-}}=-\lambda \alpha w_{ij}e^{\frac{-\left|\Delta s\right|}{\tau }}\text{ if}\Delta s<0 \end{array}$$ (7)

With *μ* = 0.4, *w*_*0*_ = 5% of mean initial weights (*w*_*ij*_(0)), *λ* = 0.1, *α* = 0.11, *τ* = 20 ms [58], and here Δs denotes the delay between vesicle exocytosis and the postsynaptic action potential, rather than the integration time step in the preceding text.

We use homeostatic scaling to maintain the overall firing rate of the postsynaptic neuron at a fixed level across longer periods [57]. This results in competition because strongly activated synaptic connections undergo potentiation at the expense of other synaptic connections projecting to the same postsynaptic neuron, balancing the total incoming synaptic activity to maintain the desired output firing rate [63]:

$$\tau_{h}\frac{dw_{ij}}{dt}=(r_{m}-r_{j})\cdot w_{ij}$$ (8)

With *τ*_*h*_ the timescale of homeostatic scaling, *r*_*m*_ the desired firing rate (which we set to the mean input firing rate) and *r*_*j*_ the firing rate of postsynaptic neuron *j* (the running average is across the last 12 spikes). For parameter values see Table 1.

The network consists of 500 input neurons that are connected to 10 output neurons. A subset of neurons that has high firing rates represent neurons with receptive fields that are strongly activated by external stimuli. The presynaptic neurons produce Poisson distributed action potentials according to a probability p_s_ = r_i_ * Δt. Initially, SVE equilibrates the network to the desired firing rate (*r*_*m*_), leading to a mean synaptic weight *w*_*ij*_ *(0)*. Synaptic strength has hard bounds at 0 and 8 * *w*_*ij*_(0), when it exceeds these extreme values it is reset to 0 or 8 * *w*_*ij*_(0), as appropriate.

Learning has occurred when the distribution of synaptic weights has become bimodal. We quantified the learning rate as the time change of the ratio of the median synaptic weight of the higher firing rate synaptic connections (*M*_*high*_) over that of the lower firing rate synaptic connections (*M*_*low*_):

$$\Lambda (t)=\frac{dD(t)}{\Delta t}$$ (9)

Here *Λ(t)* is the learning rate, *D(t) = M*_*high*_ *(t) / M*_*low*_ *(t)*. We used median synaptic weights because it is more robust than the mean and we used a ratio to make the measure independent of the overall scale of *M*.

To mimic a biological developmental profile, we used mRNA expression levels for Doc2b (SVE), Doc2a (aEVE) and Synaptotagmin1 * Complexin1 (sEVE) from a database of experimentally measured mRNA expresssion levels [31]. During maturation, in the model new synapses were formed with strength *w*_*ij*_ and probability (P):

$$P_{ij}=k\cdot \Delta t$$ (10)

New synapses form between unconnected neurons *i* and *j* at a rate of 8% newly formed synapses per 2 weeks for early development (k = 6.6 x 10^−8^), 5% during early childhood (k = 5.0 x 10^−8^) and 3% during adolescence and later (k = 2.5 x 10^−8^) [32]. The generation rates were increased by a factor 10^6^ for computational efficiency, while the relative duration of each developmental stage was preserved [31]. Synaptic stabilization is activity-dependent and involves the formation of PSD-95 [64], and weak synapses are in general easily pruned [33]. Hence, weak synapses with strength *w*_*ij*_ < 0.7 *w*_ij_, were eliminated and large synapses were limited to strength 8 * *w*_ij_.

### Competitive rate model

We used a rate model incorporating the dynamics due to vesicle-timing-dependent plasticity to study the effect of the initial weight distribution on the ability to encode patterns of action potentials. It incorporates competition between the exocytosis rates from all presynaptic neurons *i* that project to the same postsynaptic neuron *j*:

$$\frac{d}{dt}w_{ij}=\gamma (x_{ij}-{\bar{x}}_{j})\cdot s$$ (11)

Where *w*_*ij*_ is the synaptic strength, *x*_*ij*_ is the rate of vesicle exocytosis (see Eq (12) below), *$\bar{x}$*_*j*_ is the average input rate to postsynaptic neuron *j*, *γ* the rate constant and s is defined below (parameter values in Table 1). We use homeostatic scaling to maintain the overall firing rate of the postsynaptic neurons at a fixed level across longer periods (see Eq (8), [57]). To keep synaptic weights within a specific range, the rate at which weights change is scaled [65], here by choosing *s = w*_*ij*_^2^ for LTD (*x*_*ij*_ *< x*_*j*_) and *s = w*_*ij*_ *− w*_*ij*_^*2*^ for LTP (*x*_*ij*_ *> x*_*j*_). Hence large synapses undergo stronger depression during periods of little activity, whereas medium sized synapses are most strongly potentiated in times of correlated activity [66]. This scaling factor affects the overall magnitude of the rate of change of *dw*_*ij*_, but the direction of change, whether it gets weakened or strengthened, depends on the rate of exocytosis at each synapse, which is modeled as a combination of SVE and EVE:

$$x_{ij}=\xi \cdot x_{ij,sve}+(1-\xi )\cdot x_{ij,eve}$$ (12)

Where ξ is the contribution of SVE (*x*_*ij*,*sve*_) and 1 − ξ being the contribution of EVE (*x*_*ij*,*eve*_). The EVE distribution for *x_ij_* is bimodal, with a subset of presynaptic neurons having high rates *(x*_*ij*,*eve*_ = 0.8) and other neurons fire at a lower rate (*x*_*ij,eve*_ = 0.4). The rate *x_ij_* for SVE is taken from a Gaussian distribution (*μ* = Σ*x*_*ij*,*eve*_ / N^2^) and standard deviation 0.05. The initial synaptic weights are either unimodal Gaussian distributed (*μ ±* 0.05) or bimodal (*μ*_*weak*_ = 0.2 ± 0.02, *μ*_*strong*_ = 0.8 ± 0.08). To quantify the degree to which weights represent the pattern of presynaptic spiking, we calculate the overlap between the resulting weights and the applied patterns of action potentials using the Heaviside step function (Θ):

$$P=\frac{1}{N^{2}}\sum_{i,j=1}^{N}\left(2\Theta [w_{ij}-\bar{w}]-1)\cdot (2\Theta [x_{ij}-{\bar{x}}_{0}]-1\right)$$ (13)

This measures the fraction of synapses with a higher than average strength that belong to a presynaptic neuron with a higher than average activity. Here *w*_*ij*_ and *x*_*ij*_ are the synaptic weight and vesicle exocytosis rate from presynaptic neuron i to postsynaptic neuron j, respectively. The thresholds inside the Heaviside step functions are the average synaptic weight and average presynaptic spiking *x*_*0*_. The overlap between the presynaptic spiking and synaptic weights is normalized by the total number of synapses (*N* ^*2*^). If the synaptic weights correlate perfectly with presynaptic spiking, *P* will attain its maximal value of one. If the weights are uncorrelated with the presynaptic spiking, *P* will approach zero for large enough networks.
